# Editorial: Calcium signaling: an early plant defense response against pests and pathogens

**DOI:** 10.3389/fpls.2024.1400006

**Published:** 2024-03-28

**Authors:** Archana Singh, Dhandapani Gurusamy, Indrakant K. Singh

**Affiliations:** ^1^ Department of Plant Molecular Biology, University of Delhi, New Delhi, India; ^2^ Department of Botany, Hansraj College, University of Delhi, Delhi, India; ^3^ Delhi School of Climate Change and Sustainability, Institution of Eminence, Maharishi Karnad Bhawan, University of Delhi, Delhi, India; ^4^ Department of Botany, Kongunadu Arts and Science College (Autonomous), Bharathiar University, Coimbatore, Tamil Nadu, India; ^5^ Molecular Biology Research Lab, Department of Zoology, Deshbandhu College & Delhi School of Public Health, Institution of Eminence, University of Delhi, Delhi, India

**Keywords:** Ca^2+^ signaling, cyclic-nucleotide-gated channels (CNGCs), calmodulin, biotic stress, crop production

Plants are exposed to biotic stresses caused by interactions with other living organisms. This leads to adverse effects on their growth, development and productivity. Plants have evolved sophisticated defense mechanisms to protect themselves, including sensing biotic cues, signal transduction, reprogramming at transcript, protein as well as metabolite levels to strengthen their defense status. One important macronutrient for plants is calcium, which plays a significant role in the early signaling pathways that govern plant-biotic interactions. Plants produce a calcium signature in response to pest or pathogen attack, which acts as a signal. In order to activate the defense mechanism, these signals are detected by calcium sensors and then sent on to downstream signaling components. Our comprehension of the biochemical and molecular elements of calcium signaling, such as Calmodulin (CaM), CaM-like proteins (CML), Calcineurin B-like proteins (CBL), Calcium dependent protein kinases (CDPKs) and their transporters viz Cyclic nucleotide gated channels (CNGCs), two pore channels (TPCs), Annexins, Glutamate-like receptor channels, Ca^2+^/cation exchangers (CCXs), Ca^2+^-ATPases, Ca^2+^/H^+^ exchangers (CAXs), has advanced recently. Even though a number of cutting-edge studies have been carried out, little is known about the decoding of the full components of the calcium signaling pathway and how it interacts with other relevant pathways, such as the Reactive Oxygen Species (ROS) and Mitogen-Activated Protein Kinase (MAPK) pathways, when a pathogen and pest interact. Through genome editing and genetic engineering, scientists will be able to modify the calcium signaling system and its components, which are essential in plant defense, to create plants that are more resistant to pests and diseases.

In the present Research Topic, Neelam et al. highlighted the critical involvement of calcium signaling pathways in plant responses to harmful and helpful microorganisms, illuminating the complex dynamics of these interactions. Defense signaling systems are triggered and relayed by modulating calcium signaling, especially by microbial effectors in pathogenic environments. Since many different Ca2+ binding and decoding proteins are involved in the complex process of Ca2+-dependent signaling, it is important to identify the genes that control the Ca2+ surge, especially when it comes to agricultural plants. Additionally, authors stress the importance of calcium signaling in establishing symbiotic partnerships between plants and beneficial bacteria, particularly in the development of arbuscular mycorrhizal (AM) and rhizobia interactions. For nutrient uptake, stress resistance, and general plant health, this relationship is essential. It has been proposed that calcium-based fertilizers could be useful instruments to improve these symbiotic relationships and eventually increase crop yields.

Similarly, Bhar et al. evaluated the importance of the Calcium signaling pathway, emphasizing its critical function in the signal transduction machinery and its significance as a pivotal event in pathogenic plant biotic interactions. A praiseworthy feature of the research is how well the authors have explained the notion of “calcium signature”. The paper focuses on the interactions between various signaling pathways rather than just explaining the calcium signaling pathway. This inclusion is essential because it clarifies how different routes are connected to one another, which eventually helps to establish an efficient and natural resistance response. The authors shed important light on the holistic nature of plant defense mechanisms by expanding on this crosstalk. The work recognizes the complex relationship between ROS and MAPK with calcium signaling and addresses this relationship correctly. This study’s finding highlights the dynamic character of signal transduction networks and adds to the complexity of plant defense mechanisms.

Furthermore, Hamouzová et al. suggested and reported the role of Ca2+ signaling in weeds stressed by herbicides. The authors investigate the potential sensing and molecular communication via Ca2+ signals in weeds under such stress conditions, and they suggest that Ca2+signaling may result in transcriptional reprogramming during herbicide stress. Stress-responsive genes like GSTs and CYP450s are overexpressed in weeds that are resistant to herbicides. The authors put forth a theory on the potential for herbicide resistance to be conferred by a Ca2+-mediated signal network. The scientists point out how important it is to keep an eye on Ca2+ signal patterns linked to herbicide stress responses and speculate that weeds may be affected by Ca2+ signal-regulated alternative splicing. Herbicide processes and the herbicide stress signaling network remain poorly understood, despite advances in our knowledge of Ca2+-mediated signal networks in crops and plants under a variety of environmental stressors. To clarify the roles of Ca2+ signaling in weeds under herbicide stress, the authors call for additional research efforts that include obtaining reference weed genomes, RNA-seq transcriptome studies, modelling approaches based on bioinformatics, and gene editing experiments (using RNAi and/or CRISPR/Cas systems).

Additionally, calcium cyanamide (CaCN2) has been shown by Xie et al. to have the potential to be a dual-purpose agricultural agent that serves as a soil cleanser and fertilizer. The capacity of CaCN2 to reduce Corynespora blight and prevent the establishment of pathogens in soil derived from plant debris are indications of its fungicidal activity. Furthermore, one reason for the soil’s efficacy in suppressing soil-borne pathogens is the conversion of CaCN2 into hydrogen cyanamide. The fact that CaCN2 lessens pathogens’ prevalence in soil residues rather than totally eliminating them is one noteworthy finding of this study. With its ability to prevent soil-borne pathogens as well as soil wastes, CaCN2 may be an important factor in controlling plant diseases and preserving the health of the soil. Additionally, it has been found that the combination of CaCN2 and polyethylene film (PE) treatment—specifically, PE—is more successful in preventing the transfer of disease residues from soil to the atmosphere. This data implies that a major decrease in cucumber leaf disease may result from the integration of these technologies into agricultural operations, particularly in the growing of protected vegetables.

Plants frequently face both biotic and abiotic stresses at the same time, and a crucial component of their survival and adaptability is how they sort and combine these cues. Xu et al. examined the integration of various stress conditions in signaling responses and the function of calcium (Ca2+) signals in sensing abiotic environmental cues while taking into account the various pressures placed on plants. Abiotic stresses include elements like temperature, salt, drought, and other environmental variables, whereas biotic stresses are caused by living things like pathogens and pests. The report explores new developments that provide insight into the processes by which plants use Ca2+ signaling to sense and react to abiotic stressors. Developing solutions to improve crop stress tolerance requires an understanding of these systems, which is crucial given the influence of climate change on global agriculture.

This compilation highlights the importance of Ca2+ signaling during plant-biotic interactions and adds to our understanding of complex signaling networks. With a deeper comprehension of Calcium signaling and the elements of the plant defense pathway, scientists will be able to use genome editing and genetic engineering to modify the process and increase plant resistance to pests and diseases. The integration of the detection of various stress situations and the ensuing signaling reactions that require attention are also included in this Research Topic. This information may facilitate the creation of focused methods to control calcium signaling for better crop protection and strengthened symbiotic connections.

## Author contributions

AS: Conceptualization, Data curation, Writing – review & editing. DG: Writing – review & editing. IS: Writing – review & editing.

